# Longitudinal Analysis of Mental Health in Autistic University Students Across an Academic Year

**DOI:** 10.1007/s10803-022-05560-9

**Published:** 2022-07-08

**Authors:** Matthew Scott, Jenni Leppanen, Melissa Allen, Chris Jarrold, Felicity Sedgewick

**Affiliations:** 1 School of Education, University of Bristol, Bristol, UK; 2 School of Psychology, University of Cardiff, Cardiff, UK; 3 Institute of Psychiatry, Psychology and Neuroscience, King’s College London, London, UK; 4 School of Psychological Science, University of Bristol, Bristol, UK

**Keywords:** Autism, Higher education, Mental health, Longitudinal

## Abstract

**Background:**

Autistic people have worse mental health (MH) than non-autistic people. This proof-of-concept study explored feasibility of longitudinal research with autistic university students, focusing on their MH and coping styles across an academic year.

**Methods:**

Twenty-two students took part at all timepoints. They completed four rounds of online MH questionnaires.

**Results:**

Over 80% of students were retained. They started the year with high levels of all MH issues, which remained stable across the year. Network Change analysis showed the connections between MH and coping style changed over time.

**Conclusions:**

Autistic students are engaged participants who are likely to take part in longitudinal research. While MH levels were stable, it may be that coping styles are a useful target for intervention.

In the general UK population, up to one in four adults will experience mental health problems at any one point in time (McManus et al., 2016), and these issues often develop around the transition out of childhood (Jones, 2013). There is a wealth of research suggesting that autistic people experience worse mental health than the general population across the lifespan – from childhood anxiety (White et al., 2009) to adult depression and suicidality (Cassidy & Rodgers, 2017), with up to 80% of autistic people facing at least one of these challenges (Lever & Guerts, 2016). These mental health difficulties are associated with significantly lower subjective quality of life in autistic adults (Mason et al., 2019).

Anxiety and depression are the most commonly diagnosed mental health issues among autistic people, with between 40 and 80% of adults meeting clinical criteria (Lugo-Marín et al., 2019). Eating disorders (EDs) are also common in both the general population, at around 1% (Nicely et al., 2014), and autistic populations (disordered eating is reported by around a third of autistic people, Sedgewick et al., 2020). Obsessive Compulsive Disorder (OCD) is also more common among autistic than non-autistic people, though this is partly due to overlap in the diagnostic checklists (e.g. preference for order/sameness). Despite this overlap, a systematic review indicated that around 17% of autistic people could be additionally diagnosed with OCD (van Steensel et al., 2011). It is estimated that 30–50% of autistic children also meet criteria for Attention Deficit Hyperactivity Disorder (ADHD) (Davis & Kollins, 2012). It is likely that those individuals who have both diagnoses are especially vulnerable to mental ill health (Daviss, 2008; Tsang et al., 2015). All these issues are complicated by a lack of appropriate and tailored professional support, something which has been highlighted by autistic young people (Crane et al., 2019).

Some of the ways in which non-autistic people cope with poor mental health are well known – turning to alcohol and drugs to self-medicate is common, and there are a range of recognized psychological coping mechanisms (e.g., humour, reframing, self-blame) (Fletcher & Sarkar, 2013). While research into alcohol consumption among autistic people is only just beginning, it has been shown that they use it in similar ways to non-autistic people, especially to mitigate social anxieties (Brosnan & Adams, 2020). Further, preliminary research evidence suggests that there is an increased risk of substance use problems in autistic people relative to non-autistic people (Butwicka et al., 2016). However, more recent research has suggested that this may be more related to autism-specific expectations of the effect of alcohol, such as increased social and communication skills when drinking (Brosnan & Adams, 2020). Regarding recreational use of illegal substances, the limited evidence suggests that autistic people are less likely to experiment than their non-autistic peers (Rengit et al., 2016). Psychological coping mechanisms among autistic people have also not been extensively investigated, though a recent paper suggested that avoidant coping (denial, self-distraction) is associated with worse mental health outcomes for autistic adults (Muniandy et al., 2020).

The above review summarises a wealth of work on the mental health experiences of autistic people. However, this work has tended not to examine the mental health of specific groups of autistic people, such as autistic university students. Research over many decades has shown that non-autistic university students often struggle with novel mental health challenges as they navigate this time of physical, social, and personal transition (Briggs et al., 2012).

It is known that among the non-autistic student population, mental health fluctuates over the academic year. For example, reported distress peaks in Semester One, then decreases – although it tends to remain higher than that reported pre-university (Bewick et al., 2010). Similarly, depression has been shown to be highest at the end of Semester One for first year students and to decrease over the rest of the academic year, though with localized spikes in response to periods of increased workload stress (Barker et al., 2018). Many students report increasing anxiety and depression over Years Two and Three, as workload stress builds up to their final degree classification (Macaskill, 2013).

Coping mechanisms have been shown to have an impact on these generalized patterns of student mental health. Those who report using ‘approach’ strategies (positive reframing, planning) showed decreases in anxiety and depression from Semester One to the end of the academic year, whereas those using ‘avoidant’ strategies showed an increase in both over the same period (Stewart et al., 1997). This emphasizes the importance of understanding coping alongside mental health difficulties, as it has an instrumental role in how mental health develops and is managed over time.

The experiences of autistic students in higher education have only recently become a focus of research interest, partly due to an historical assumption that autism was in some part synonymous with intellectual disability, and therefore that few autistic children would perform well enough academically to attend. For autistic students, who may find such transitions inherently more difficult and distressing (Cheak-Zamora et al., 2015), mental health problems may further intensify at this time. Indeed, conditions such as anxiety and depression have been shown to be especially common among autistic people deemed ‘cognitively able’ (Strang et al., 2012) – a group encompassing those who are most likely to attend university.

Currently the existing literature suggests that, just as in the broader population, autistic students have worse mental health than their non-autistic counterparts. A recent review paper found that autistic students are roughly three times more likely to report having at least one mental health condition than non-autistic students, and that anxiety and depression were most common among these conditions with between 31% and 90% of students naming one or both issues (Kuder et al., 2020). This is despite these individuals being academically capable of university level study (Jackson et al., 2018).

The drivers behind these difficulties are multifaceted. Elements of autistic people’s sensory, cognitive and emotional functioning differences may contribute to difficulties at university, as they face increased responsibility for planning their time, new sensory environments, and a wide range of social situations (South & Rodgers, 2017). These factors may make the transition to university particularly difficult for autistic students (Toor et al., 2016). Social factors are consistently named as key in autistic student’s university experiences, both positive and negative – many autistic students want to be involved in the social life of their institution, but face barriers accessing this (Gurbuz et al., 2019). Many studies have emphasized that autism stigma and a lack of autism knowledge, especially among academic and pastoral staff, has a negative impact on autistic students’ mental health as it makes them less likely to seek support, and less likely to get appropriate support when they do reach out (Scott & Sedgewick, in press; Vincent et al., 2017). This can make ‘dropping out’ the only feasible option for some autistic students (Cage & Howes, 2020), and autistic students are less likely to graduate from post-secondary education than their non-autistic peers (Newman et al., 2011).

Understanding which mental health issues are most common among autistic students, and how these vary across an academic year, can help higher education institutions to best support these students. The lack of longitudinal work with autistic students is a significant gap in the literature, as we would expect any range of measures to change over time, especially among individuals settling into a new environment and transitioning to independence. This study, then, functions partly as a proof-of-concept vehicle to test the interest in, and retention levels for, autistic students to take part in longitudinal research while at university. Additionally, given the above evidence from the literature, the current study sought to explore the ways in which a group of autistic student’s mental health – and the coping mechanisms they employed – fluctuated across an academic year. The research questions were:

1. What are the levels of common mental health issues among autistic university students?
2. Do the levels of these mental health issues change over the course of an academic year?
3. What coping mechanisms do autistic university students employ, at what levels, over the course of an academic year?
4. How coping style impacts mental health and whether this relationship changes over time?
5. What is the retention of autistic students in a longitudinal study over an academic year?

## Methods

### Participants

Inclusion criteria were that participants (1) had an autism diagnosis and (2) were a current University of Bristol student (the institution where the research was carried out). Individuals responded to advertisements put out over social media, direct emails from university departments/services, poster advertisements around campus or heard about the study via word-of-mouth. The School of Education’s ethics committee within UOB granted the study ethical approval, and all participants gave informed consent before taking part.

Twenty-seven autistic students volunteered and completed questionnaires at Time 1 (October 2019). Twenty-six took part at Time 2 (December 2019), 24 at Time 3 (February 2020), and 22 at Time 4 (May 2020). This represents an 81% participant retention rate, which is highly successful – especially considering the length of time involved, and that the COVID-19 pandemic occurred during data collection, which disrupted many participants lives. There were no apparent differences in age or other factors between those who dropped out during the process and those who remained involved – those who dropped out were mostly female (four of five) and mostly undergraduates (two first-years, one second-year, one third-year, and one Masters student). All were of White British ethnic origin. Those who dropped out had similar MH scores to those who completed all rounds in terms of anxiety, depression, social anxiety, ADHD and OCD, and their behavioural scores (alcohol use, drug use) were also similar. Statistical tests comparing participants who were retained with those who dropped out were not carried out due to the small numbers of participants in the latter group.

All participants who completed all rounds of questionnaires were included (*n* = 22). This constituted a sample of 13 female individuals, 8 male individuals and 1 nonbinary individual, with an age range from 19 to 36 years at T4 (*Mean* = 21.31, *SD* = 4.19), all of whom reported having a formal clinical diagnosis of being on the autism spectrum, said diagnosis being the basis of Disability Support Statements for all participants. The sample was majority White British (18 participants), with two participants identifying as White other, one as Mixed ethnicity, and one as Latino. Sixteen participants were undergraduates (nine first-years, seven second-years), four were Masters/PGCE students, and two were PhD students (one second-year, one third-year). Nine participants were in Science, Technology, Engineering and Mathematics departments, and thirteen participants were in Art, Humanities and Social Sciences departments. Fourteen participants disclosed at least one existing mental health diagnosis (most common were anxiety = 12, and depression = 10), with ten participants having two or more mental health diagnoses.

*Procedure*.

The study adopted an observational longitudinal design, with online questionnaires completed at four time-points (T1-T4) across the academic year. At T1, participants provided demographic information and were asked for changes to this at further time points. Participants were emailed a link to the study page and were paid £20 in Amazon vouchers per round of participation – each of which took 20 to 30 min. The study took place during the emergence and early stages of the COVID-19 pandemic, which undoubtedly would have had a significant impact on our participants. However, because the research had always been designed to be conducted online, our procedures did not have to change in response to the pandemic.

### Measures

*SCID Depression screening* (First, 2015): The SCID is a widely used clinical screening measure, asking participants to report the frequency of depression-related cognitions and behaviours, rated from 0 to 3 (e.g. ‘I do not feel sad’ to ‘I am so sad and unhappy that I can’t stand it’). The 21-item measure exhibits good validity and reliability (Shankman et al., 2018).

#### General anxiety disorder questionnaire (GAD-7; Spitzer et al., 2006)

The GAD-7 asks participants to report the frequency of anxiety-related difficulties. The seven-item measure exhibits strong psychometric properties within the general population (Spitzer et al., 2006), and high internal consistency within an autistic sample (Hull et al., 2018).

*Mini Social Phobia Inventory (Mini-SPIN; Connor et al., *2001): The Mini-SPIN focussed on participants’ level of agreement with statements related to social phobia. Validity and reliability have been established with non-autistic samples (Connor et al., 2001; Wiltink et al., 2017), and descriptive data suggests that the Mini-SPIN has potential as a screening tool for autistic people (Nah et al., 2018).

*SCOFF Questionnaire (Morgan et al., 1999)*: Participants answered five ‘yes/no’ questions covering the core behaviours of anorexia and bulimia nervosa. The scale has been validated with university students (Garcia et al., 2010), and showed good reliability with autistic women (Sedgewick, 2018).

#### Adult ADHD self-report scale (ASRS-v1.1 - Kessler et al., 2005)

Participants were asked to rate how often they had recently encountered difficulties related to ADHD. The scale has strong internal and concurrent validity (Adler et al., 2012), and is accurate for detecting ADHD-specific symptoms amongst people with clinical depression (Dunlop et al., 2018).

#### Zohar-Fineberg Obsessive compulsive screen (Z-FOCS; NICE, 2005)

This scale contains five ‘yes/no’ questions about obsessions and compulsions. The measure is sensitive to symptoms and displays adequate reliability and specificity (Fineberg et al., 2008).

#### Alcohol Use Disorders Identification Test (AUDIT - Saunders et al., 1993)

The AUDIT centres on how often - over the last year (T1), or since the last set of questionnaires (T2-T4) - participants engaged in varyingly extreme drinking activity. Psychometric properties of the AUDIT are favourable when compared to other self-report measures of disordered alcohol use (Reinert & Allen, 2007).

#### Drug abuse screen test (DAST-10 - Skinner, 1982)

Participants answered ten ‘yes/no’ questions about drug-taking habits over the last year (T1), then since the last set of questionnaires. The DAST-10 exhibits good specificity and sensitivity amongst individuals with other mental health diagnoses (Maisto et al., 2000).

#### Brief coping orientation to problems experienced questionnaire (Brief-COPE; Carver, 1997)

The Brief-COPE was completed at T2-T4 (Dec 2019, Feb 2020, May 2020). Participants made judgements on a four-point Likert scale regarding how often they employ 14 different approaches to coping with hardship. These sub-scales can be grouped under two validated and reliable coping styles – ‘avoidant’ (such as denial, substance use, and self-blame) and ‘approach’ (such as positive reframing, acceptance, and planning) (Eisenburg et al., 2012).

### Community Involvement Statement

This study was actively carried out with members of the autism community. Autistic consultants, including an autistic university student, were involved in the funding application and research questions, design of the study, checked measures for accessibility and reviewed the manuscript presenting our findings. Following T1 (Oct 2019), we had further discussions with this group and some participants, and then decided to include the Brief-COPE at all other timepoints as the initial battery had been less demanding for participants than we had anticipated.

### Data Analysis

All statistical analyses were conducted with R version 4.0.3 (R Core Team, 2018). T1 levels of each mental health condition were calculated, then compared to each subsequent time point. As the COVID-19 global pandemic took hold between T3 and T4, we explored whether T4 was statistically significantly different to other timepoints and found that it was not on all constructs (all *ps >* 0.05), bar depression (*p =* 0.036) and social anxiety (*p* = 0.048). These data were therefore retained in all further analyses, and the change in depression scores is discussed below. Linear mixed effects models from the package *lme4* (Bates et al., 2015) were used to examine impact of coping style and changes over time in each mental health variable. In the case of count variables, alcohol and drug use, a Poisson family generalised linear mixed effects model was applied. In an attempt to reduce dimensionality of the data, a single coping score was created by subtracting each participant’s avoidance style score from their approach style score. This resulted in a single coping score where positive values indicated greater general tendency to use approach strategies and negative values indicated greater general tendency to rely on avoidance. In the linear mixed effects models the coping score, time and coping by time interaction were entered as predictors. This approach enabled us to examine changes in mental health and alcohol and drug use over time, the impact of coping style, and whether the impact of coping style varied as function of time. Threshold for statistical significance was set at p < 0.05 and Bayes Factors (BF) were calculated using the *BayesFactor* package to quantify the evidence in favour or the alternative hypotheses. BF > 1 was interpreted as providing evidence in favour of the alternative hypothesis, BF = 1 favoured neither the alternative nor the null hypothesis, and BF < 1 favoured the null hypothesis.

## Results

### Mental health and coping style

T1 data suggested that there were high levels of mental health issues among our participants, with the mean score being above clinical cutoffs for depression, anxiety, social anxiety, ADHD, and OCD. Descriptive statistics for each mental health measure at each time point are presented in Table 1.

**Table 1:** *Descriptive statistics for mental health at each timepoint*

	T1 Range Mean (SD)	T2 Range Mean (SD)	T3 Range Mean (SD)	T4 Range Mean (SD)
Depression (max = 63, cutoff = 15)	1–40 17.08 (9.39)	1–40 17.27 (11.32)	0–36 16.05 (8.92)	1–46 20.05 (11.71)
Anxiety (max = 21, cutoff = 10)	1–21 10.08 (5.75)	0–24 12.00 (7.12)	1–21 10.91 (7.14)	2–23 12.68 (7.45)
Social anxiety (max = 68, cutoff = 30)	17–55 37.20 (12.05)	6–59 36.64 (14.53)	10–59 35.05 (14.72)	11–60 34.09 (14.53)
Anorexia and bulimia (max = 5, cutoff = 2)	0–5 1.48 (1.53)	0–3 1.27 (1.12)	0–4 1.36 (1.29)	0–5 1.23 (1.41)
ARFID (max = 18, cutoff = 5)	0–12 3.72 (2.82)	0–11 4.32 (3.26)	0–8 3.32 (2.17)	0–11 4.23 (2.96)
ADHD (max = 24, cutoff = 6)	1–22 13.76 (5.45)	0–23 14.32 (6.24)	4–22 12.45 (4.76)	5–19 14.32 (4.49)
OCD (max = 6, cutoff = 3)	0–6 3.32 (1.89)	0–5 3.41 (1.44)	1–6 3.32 (1.36)	0–6 3.59 (1.89)
Alcohol use (max = 40, cutoff = 8)	0–11 3.40 (3.92)	0–13 3.18 (4.41)	0–9 2.45 (3.17)	0–9 1.45 (2.26)
Drug use (max = 10, cutoff = 6)	0–8 1.68 (2.08)	0–7 1.41 (1.59)	0–7 1.32 (1.43)	0–6 1.50 (1.54)

*NB*: Only four drug users

The BRIEF-COPE was introduced at T2. For each timepoint, levels of each coping strategy and summary statistics for ‘Approach’ and ‘Avoidant’ styles, are presented in Table 2. T2 data suggested that Self-Blame, Self-Distraction, and Acceptance were the most common forms of coping strategy employed by autistic students when dealing with stressful events. Substance use, Religion and Denial were the least commonly used coping strategies.

**Table 2:** *Descriptive statistics for BRIEF-COPE*

Coping Style (all max = 8)	T2 Range Mean (SD)	T3 Range Mean (SD)	T4 Range Mean (SD)
Self-distraction (Av)	2–8 5.18 (1.79)	2–8 5.45 (1.87)	2–8 5.14 (1.83)
Active Coping (App)	3–8 4.55 (1.47)	2–8 4.91 (1.80)	2–8 4.86 (1.36)
Denial (Av)	2–8 2.77 (1.54)	2–7 2.73 (1.52)	2–8 3.27 (1.49)
Substance Use (Av)	2–8 2.55 (1.44)	2–6 2.45 (1.06)	2–4 2.18 (0.59)
Use of Emotional Support (App)	2–8 4.73 (1.61)	2–8 4.77 (1.66)	2–8 4.27 (1.70)
Use of Instrumental Support (App)	0–8 4.32 (1.84)	2–8 5.00 (1.80)	2–8 4.14 (1.91)
Behavioural Disengagement (Av)	2–8 3.91 (1.77)	2–8 3.36 (1.43)	2–6 3.64 (1.26)
Venting (Av)	2–8 3.86 (1.46)	2–7 4.09 (1.38)	2–6 3.95 (1.25)
Positive Reframing (App)	2–7 4.05 (1.46)	2–8 4.36 (1.73)	2–8 4.00 (1.60)
Planning (App)	2–7 4.73 (1.39)	2–8 4.82 (1.84)	3–7 4.64 (1.18)
Humour (Ambiguous)	2–8 4.64 (2.15)	2–8 4.64 (2.15)	2–8 4.23 (1.74)
Acceptance (App)	2–8 4.91 (1.57)	2–8 5.05 (1.43)	2–8 4.64 (1.65)
Religion (Ambiguous)	2–5 2.73 (1.03)	2–8 2.77 (1.48)	2–7 2.95 (1.46)
Self-Blame (Av)	2–8 5.82 (2.02)	2–8 5.64 (2.24)	2–8 5.14 (2.12)
Approach	2.33–6.83 4.55 (1.02)	2.83–7.67 4.82 (1.24)	3.17–6.33 4.42 (0.92)
Avoidant	2.17–6.17 4.02 (1.03)	2.17–6.17 3.97 (0.96)	2.17–5.33 3.89 (0.83)

### Mental health and coping style over time

Prior to conducting the linear mixed models with coping style as one of the predictors we examined whether there were any significant changes in coping style over time. If coping style varied as function of time including both variables as predictors could introduce multicollinearity in the linear mixed models. There were strong correlations between all Approach strategies across all timepoints (*ps* < 0.07), and similarly strong correlations between all Avoidant strategies across all timepoints (*ps* < 0.05). Therefore, to reduce the number of tests conducted on a small sample, we did not run tests for each strategy within each overarching style. There was a main effect of coping style, with Approach being used more than Avoidant (*F(1,105)* = 18.96, *p* < 0.001, BF = 775.66 ± 2.14) with the BF providing strong evidence in favour of this result. There was no main effect of time on coping (*F(2,105)* = 0.51, *p* = 0.60, BF = 0.14 ± 1.56) and no time by coping style interaction (F(2,105) = 0.51, p = 0.603, BF = 0.18 ± 6.04) with the BFs providing moderate evidence in favour of the null hypothesis. This suggests that coping style could be introduced as a predictor alongside time to examine the impact of these two variables on mental health and alcohol and drug use.

That analysis showed that there was a significant effect of coping on Depression (F(1,52) = 18.30, p < 0.001, BF = 721.44), Anxiety (F(1,48) = 7.20, p = 0.010, BF = 8.43), Social Anxiety (F(1,44) = 6.88, p = 0.012, BF = 5.75), and ARFID scores (F(1,51) = 6.90, p = 0.011, BF = 7.48). In all cases lower coping scores, indicating increased reliance on avoidance, was associated with more mental health difficulties. The corresponding BFs indicated that there was moderate to very strong evidence in favor of the alternative hypotheses. There was no significant effect of coping on Anorexia/Bulimia scores (F(1,47) = 2.32, p = 0.135, BF = 0.59), ADHD scores (F(1,48) = 1.01, p = 0.319, BF = 0.83), OCD scores (F(1,53) = 1.70, p = 0.197, BF = 0.54), Alcohol use (X^2^ (1) = 1.89, p = 0.169, BF = 0.20), or Drug use (X^2^ (1) = 0.07, p = 0.787, BF = 0.13). The associated BFs provided anecdotal to moderate evidence in favor of the null hypothesis suggesting the present data were 1.2 to 7.9 times more likely under the null hypothesis.

There was a main effect of time for Alcohol Use (*X*^*2*^*(2)* = 12.90, *p* = 0.002, BF = 22.64) with the BF providing moderate evidence in favour of this result and suggesting the data were approximately 22.6 times more likely under the alternative hypothesis (see Fig. 6). Post-hoc tests revealed that this was driven by a significant decrease in Alcohol Use between T2 and T4, *z* = 3.37, *p* = 0.002. There was no main effect of time for Depression scores (*F(2,39)* = 2.36, *p* = 0.107, BF = 0.50), Anxiety scores (*F(2,40)* = 0.66, *p* = 0.521, BF = 0.28), Social Anxiety scores (*F(2,40)* = 1.66, *p* = 0.203, BF = 0.25), Anorexia/Bulimia scores (*F(2,40)* = 0.22, *p* = 0.894, BF = 0.15), ARFID scores (*F(2,41)* = 1.26, *p* = 0.295, BF = 0.50), ADHD scores (*F(2,40)* = 1.12, *p* = 0.337, BF = 1.1), OCD scores (*F(2,41)* = 0.69, *p* = 0.508, BF = 0.16), or Drug Use (*X*^*2*^ (2) = 1.32, *p* = 0.501, BF = 0.02). The BF associated with all but the ADHD scores provided anecdotal to strong evidence in support of the null hypothesis and suggested that the data were 2.0 to 58.0 times more likely under the null hypothesis. Interestingly, the BF associated with the ADHD scores pointed towards anecdotal evidence for the alternative hypothesis. This suggests that overall, drug use and most mental health variables remained stable across the course of the study.

There were no significant coping by time interactions for Depression scores (F(2,44) = 1.04, p = 0.360, BF = 0.17), Anxiety scores (F(2,43) = 0.05, p = 0.950, BF = 0.12), Social Anxiety scores (F(2,41) = 0.83, p = 0.442, BF = 0.28), Anorexia/Bulimia scores (F(2,42) = 2.02, p = 0.146, BF = 0.83), ARFID scores (F(2,44) = 0.07, p = 0.928, BF = 0.12), ADHD scores (F(2,43) = 0.32, p = 0.727, BF = 0.21), OCD scores (F(2,46) = 0.76, p = 0.475, BF = 0.30), Alcohol use (X^2^ (2) = 1.77, p = 0.413, BF = 0.13), or Drug use (X^2^ (2) = 1.94, p = 0.379, BF < 0.0001). The associated BFs provided anecdotal to strong evidence in favor of the null hypothesis.

## Discussion

This study is the first to track the mental health of a group of autistic university students over the course of an academic year. Importantly, the emergence of COVID-19 and the accompanying restrictions resulted in very few participants dropping out of the study (just five of those originally recruited), suggesting excellent longitudinal participant retention rates are possible for research of this kind with autistic students. We were able to examine whether this global pandemic had an impact on autistic student mental health. Interestingly, it appeared to have relatively little impact overall, with no significant worsening of any specific measure, although it should be noted that this may be because our participants went into the pandemic with poor mental health. The one thing that changed was a reduction in alcohol consumption, possibly due to a decrease in the number of social opportunities where students would often drink together before lockdown. Interestingly, we also found that greater reliance on avoidance-based coping was associated with more depression, generalised anxiety, social anxiety and ARFID symptoms and these associations were not influenced by time.

This lack of impact of COVID-19 emphasises the overall message of stability over time in the mental health status of our participants. Autistic students arrive at the university (or at the start of a new year, for those continuing on courses) with poor mental health, in line with other research identifying high levels of depression and anxiety (MacLeod et al., 2018), and especially social anxiety (Lei et al., 2020), in this population. The key novel finding of this study is that, unlike in non-autistic student populations, the levels of these conditions do not decrease over time as students settle into university life and become more comfortable with their peers over the academic year (Bewick et al., 2010). The process of adjusting to university life, which often leads to an improvement in mental health for non-autistic students, is not having the same affect for autistic students, which may create a negative cycle whereby their poor mental health makes it harder to integrate into university life, which itself contributes to maintaining poor mental health via social isolation and higher stress levels. However, a reduction in alcohol consumption over time was seen in both our autistic sample and is common in non-autistic samples, possibly due to the ‘Freshers Week’ drinking culture which is captured at Time 1 (Bewick et al., 2008).

Stability in mental health scores is seen at the individual level as well as in a lack of group differences across timepoints, suggesting that those who are struggling the most with their mental health often continue to struggle across the academic year. This suggests that autistic students are either not accessing, or not benefitting from, the mental health support available to them through the university, an issue which has been discussed previously in qualitative work (Hillier et al., 2018; McLeod et al., 2019). The persistence of mental health issues among the group, including among those who reported in interviews that they had accessed support (see Scott & Sedgewick, 2021), suggests that higher education institutions need to review their offer to autistic students, and to take on board the recommendations for tailored approaches and specific staff training which are common outcomes from research on this topic.

Interestingly, greater tendency to use avoidance as a coping mechanism was associated with worse depression, generalised anxiety, social anxiety and ARFID symptoms. These associations did not appear to change over time suggesting they were fairly stable. This finding may suggest that Avoidant coping is important in the maintenance of poor mental health, as in non-autistic groups (Stewart et al., 1997). Previous research has shown that use of Avoidant coping styles is associated with worse mental health, increased likelihood of disordered eating, and less positive outcomes for non-autistic people (Hand et al., 2017; Stewart et al., 1997). A similar finding has been shown in groups with chronic illness or disability, a category which autism and long-term mental ill health may be considered to fall into (Livneh, 2019). In support of the theory that coping styles impact long-term outcomes for students, Willoughby and Heffer (2017) showed that non-autistic first year undergraduates who employed Approach strategies had better adjustment at the end of the academic year and showed decreased suicidal ideation than those who used Avoidant strategies. It may therefore be that developing positive Approach coping strategies with autistic students would be a useful focus of intervention for university support services working with autistic students.

It may be that autistic students with poor mental health tend to utilize Avoidant coping strategies due to their existing mental health issues, which may make more proactive Approach coping strategies more mentally taxing. Avoidant coping may then lead to a reduced likelihood to seek mental health support, or greater challenges engaging with the services on offer, resulting in the continuance of poor mental health despite adapting to university life in general. This echoes the patterns seen in qualitative work around autistic student experiences with university support systems, which has described these as hard to navigate and unable to meet their specific and often unique needs (Anderson et al., 2018). Services should recognize that reaching out and asking for help can be more difficult for autistic students and find ways to make this process easier for them. Future research around supporting autistic students to develop and maintain positive coping strategies has the potential to significantly improve their experiences of university.

It is worth highlighting that our study shows that despite the challenges autistic students face at university, they are keen to contribute to research. With 81% of our participants staying engaged and completing all rounds of data collection, we can make reasonable assumptions, based on Bayesian analysis, of the likely validity of our findings of no change over time in levels of mental health difficulty. Achieving quite this level of participant retention was unexpected, considering the demands and distractions of university, and then of life during COVID. However, it is excellent news for future, larger, longitudinal studies with this population, and suggests that such work is feasible and practicable.

There are some limitations to this study. The sample is small, especially for longitudinal work, and cannot necessarily be taken as representative of all autistic students. However, our participants comprise approximately 10% of all autistic students registered at the university (as per internal figures on those students who have declared to the university that they have an autism diagnosis), a range of genders, and of study levels, which strengthens the findings. The high level of participant retention means that we have captured the mental health of all our participants across the academic year, making our study as comprehensive as possible. Furthermore, the sample is majority female, with just over half of participants identifying as such. This is potentially unexpected in autism research, considering that more males are diagnosed as autistic than females (Loomes et al., 2017). However, it has been recorded that women are generally more likely to take part in online activities, such as research, learning, and blogging, than men (Caspi et al., 2008; Pedersen & Macafee, 2007).Therefore, our sample is in line with expected patterns of engagement for the field, but is still limited in that it cannot tell us about gender differences in mental health in our sample, and may overrepresent the experiences of autistic women compared to autistic men who make up the majority of diagnosed individuals. Our research is also somewhat limited by what we chose to ask about – the range of mental health conditions and coping styles were theoretically justified, but we did not measure external (e.g. social inclusion/connectedness, assessment deadlines) or internal (e.g. camouflaging, personality traits) factors which may impact mental health. Future work should seek to include these factors in order to better understand what is influencing the reliance on avoidance coping we have seen in this study. We also acknowledge that we do not have an equivalent baseline for the coping measure as for other measures presented, as this was not collected at T1 and was a later addition to the analytic plan, in response to feedback from participants.

This proof-of-concept project is the first study to track the mental health of a group autistic university students across an academic year, highlighting the stability of mental health issues in this population – those who arrived with mental health issues did not see significant improvements, although they also did not see significant worsening of the challenges they face. This was the case despite the emergence of the COVID-19 pandemic, which is an unexpected finding – although the reduction in social anxiety makes sense considering the restrictions on meeting others and entering social spaces at university such as lectures and seminar rooms. The findings highlighted that, while there was little change in mental health over time for the students involved, coping style was strongly associated with mental health. This suggests that autistic students may acclimatise to university life over the course of the academic year, as they become familiar with their course, peers, and independent living, but that current systems do not do enough to support them in improving their mental health.
